# Subjective assessment of acute mountain sickness: investigating the relationship between the Lake Louise Self-Report, a visual analogue scale and psychological well-being scales

**DOI:** 10.1186/s40064-016-3313-z

**Published:** 2016-09-22

**Authors:** Anika Frühauf, Martin Burtscher, Elena Pocecco, Martin Faulhaber, Martin Kopp

**Affiliations:** Department of Sport Science, University of Innsbruck, Fürstenweg 185, 6020 Innsbruck, Austria

**Keywords:** Acute mountain sickness, VAS, LLS, Psychological well-being

## Abstract

**Purpose:**

There is an ongoing discussion how to assess acute mountain sickness (AMS) in real life conditions. Next to more-item scales with a cut off like the Lake Louise Self-Report (LLS), some authors suggested to use visual analog scales (VAS) to assess AMS. This study tried to contribute to this question using VAS items used for the Subjective Ratings of Drug Effects, including an additional single item for AMS. Furthermore, we investigated if instruments developed to assess psychological well-being might predict AMS assessed via LLS or VAS.

**Methods:**

32 (19 Female) adult persons with known AMS susceptibility filled in questionnaires (Feeling Scale, Felt Arousal Scale, Activation Deactivation Check List, LLS, VAS) at a height of 3650 m above sea level.

**Results:**

Correlation and regression analysis suggest a moderate to high relationship between the LLS score and the VAS items, including one VAS item asking for the severity of AMS, as well as psychological well-being.

**Conclusion:**

In conclusion, using VAS items to assess AMS can be a more precise alternative to questionnaires like LLS, for people knowledgeable with AMS. Furthermore, researchers should be aware that psychological well-being might be an important parameter influencing the assessment of AMS.

## Background

Acute mountain sickness (AMS) is common after rapid ascent to altitudes greater than 2500 m. Characteristic symptoms of AMS include headache, fatigue, insomnia, loss of appetite, nausea and vomiting (Maggiorini et al. 1990). There are currently no diagnostic markers or reliable physiological parameters to assess AMS (Hext et al. 2011). Therefore, the diagnosis of AMS depends on the self-rated assessment of symptoms’ intensity by the individual person (Kayser et al. 2010). One exception is the Hackett score which includes a structural interview and a physical examination (Hackett et al. 1976). Nevertheless, self-evaluation through questionnaires is the main way for AMS assessment (Hext et al. 2011) and the Lake Louise Self-Report (LLS) (Roach et al. 1993) is despite some critique the most popular questionnaire in use. It consists of 5 questions to be answered on a 4-point Likert scale and therefore requires little time to complete. Nevertheless, there are difficulties in the assessment of the LLS which refer to the interpretation of the terminology used (Dellasanta et al. 2007). Symptoms like headache and fatigue, as covered in the scale, may not consistently be caused by high altitude and their ratings are strongly dependent from the person asked and therefore influenced by psychological aspects. Above that, McInnis et al. (2013) revealed that sleep quality is not strongly related to other symptoms of AMS and therefore might be discussed as an implied symptom of AMS. In addition to sleep, there is an ongoing discussion about the obligatory presence of headache in the LLS. While Roach et al. (2011) underline the importance of headache as the main criterion for the diagnosis of AMS, West would rather include another assessment tool where also subjects without headache can be diagnosed with AMS (West 2011).

A different and more comprehensive way of diagnosing AMS, which might reduce difficulties in the interpretation of the wording (Roach and Kayser 2007), is the Visual Analog Scale (VAS). The VAS is known from clinical studies for measuring, among others, changes in the intensity of symptoms such as headache (Lines et al. 2001) or nausea (Hendey et al. 2005). This method uses few descriptive words and generally consists of a continuous 100 mm scale, reaching from the non-occurrence (left side) to the highest intensity of possible symptoms (right side). Participants are free to choose any point on this scale on the basis of their subjective sensation of symptoms. Wagner et al. (2007) assessed in their study AMS using VAS. They showed the VAS to have a high reliability, presenting significant correlations with the LLS. More recently, strong correlations between the LLS and VAS have been demonstrated (Hext et al. 2011; Kayser et al. 2010; Slingo et al. 2012; Wagner et al. 2012; van Roo et al. 2011). However inconsistencies in the diagnostic of AMS between those scales have been shown (Kayser et al. 2010) and no proven diagnostic cut off for AMS developed around the remark of LLS ≥ 5, has been established (Hext et al. 2011). The observed correlations were weaker when the LLS scored less than 5 (Hext et al. 2011; Kayser et al. 2010). Roach and Kayser (2007) suggested to extend the findings of Wagner et al. (2007) to different settings and languages. It is known, that hypoxia induces changes in mood states. At a height of 4300 m people became less friendly, less clear thinking, sleepier and dizzier but nevertheless happier (Shukitt-Hale et al. 1991). How the psychological well-being is related to AMS has not been evaluated yet. The following study included scales suggested for assessing psychological well-being during exercise (Ekkekakis et al. 2004) like the Feeling Scale (FS) (Hardy and Rejeski 1989), the Felt Arousal Scale (FAS) (Sevebak and Murgatroyd 1984) and the Activation-Deactivation Adjective Check List (ADACL) (Thayer 1978).

Therefore, this study aimed at answering the following questions: (1) Is there a correlation between the LLS and VAS measurement within a collective of subjects with an AMS history, during high-altitude exposure? (2) Is there a correlation between questionnaires for psychological well-being and questionnaires developed for AMS during high-altitude exposure?

## Methods

### Participants and procedure

After ethical approval from the institutional review board according to the guidelines of Helsinki, study participants were recruited by public advertisements. Subjects signed an informed consent form prior to the study. Thirty two healthy persons, 19 women and 13 men (38.8 ± 11.8 years, min: 20 years, max: 59 years; BMI 22.7 ± 2.5 kg/m^2^) volunteered for the study. At least 2 previous high-altitude exposures ≥3000 m within the last 2 years and known AMS susceptibility were inclusion criteria. 88 % of the volunteers live at a height between 500 and 700 m and 12 % between 700 and 1040 m.

The high-altitude exposure took place at the Mönchsjochhut (Jungfrau-Aletsch, Switzerland) at 3650 m for the duration of 45 h. 50 % of the subjects had a placebo and 50 % an intermittent acclimatization process in the test chamber of the university. None of the subjects knew if they were intervention or control group. For the high-altitude exposure, the subjects were driven to the bottom of the Jungfraujoch (Switzerland) at an altitude of 570 m where they spent their first night. The next morning they took the railway which carried them within 50 min to a height of 3454. The last 200 m were climbed on foot within 1 h. The volunteers slept in shared rooms and filled in the questionnaires described below during the afternoon of the second day.

### Measures

Affective valence was assessed by the Feeling Scale (FS) (Hardy and Rejeski 1989). This single-item rating scale ranges from +5 to −5, with anchors at zero (‘‘neutral’’) and at all odd integers, ranging from ‘‘very good’’ (+5) to ‘‘very bad’’ (−5). Subjects were asked to estimate their actual well-being by marking one number on the Scale. Convergent validity information for the FS has been provided (Hardy and Rejeski 1989; van Landuyt et al. 2000).

Perceived activation was assessed by the Felt Arousal Scale (FAS) (Sevebak and Murgatroyd 1984). This single-item rating scale ranges from 1 (‘‘low arousal’’) to 6 (‘‘high arousal’’). Volunteers were asked to note one number on the scale according to their actual arousal state. Arousal was explained through examples of high arousal states (anger, excitement, fear) or low arousal states (relaxation, boredom, calmness). The FAS has been used in previous physical activity studies, demonstrating convergent validity with other measures of perceived activation (van Landuyt et al. 2000).

The Activation Deactivation Adjective Check List (ADACL) (Thayer 1978) is a 20-item measure of two bipolar dimensions, namely Energetic Arousal (EA) and Tense Arousal (TA). EA extends from Energy (e.g., energetic, lively) to Tiredness (e.g., tired, drowsy), and TA extends from Tension (e.g., tense, jittery) to Calmness (e.g., calm, at rest). The ADACL was administered with its standard instructions and its 4-point response scale, which ranges from ‘‘definitely feel’’ to ‘‘definitely do not feel’’. Evidence for the reliability and structural validity of the ADACL has been provided (Thayer 1978). In previous research in the context of physical activity, the scales of the ADACL have shown satisfactory internal consistency, with values of Cronbach’s alpha coefficients ranging from .70 to .96 (Ekkekakis et al. 2005).

The VAS used in this study was developed for the purpose of subjective ratings of drug effects and called the Line Analogue Rating Scales for Sedation (LARS) (Hindmarch and Gudgeon 1980). In previous studies (Gudgeon and Hindmarch 1983; Subhan and Hindmarch 1985), 100 mm line analogue rating scales were used to assess perceived drug effects, whereas the mean score of the assessments was used as an index of subjective mood and sedation. In this study, the VAS included along the LARS items fatigue, drowsiness, dizziness, alertness and energetic, also the items relaxation and acute mountain sickness, which were rated on a 100 mm analogue line. Thereby the left side covered the non-occurrence of symptoms known in AMS (e.g. “not tired” or “not dizzy”), or the occurrence of symptoms oppositional to those known in AMS (e.g. “extremely awake”, “energetic”). The right side covered their most severe expansion (e.g. “extremely tired”, “extremely dizzy” or “not awake”, “extremely unenergetic”). The total VAS score is the mean of all accumulated items used by the VAS questionnaire.

The LLS (Roach et al. 1993) is filled in on a 4-point Likert scale (0–3) and consists of the five items headache, gastrointestinal symptoms, fatigue and weakness, dizziness and lightheadedness, and difficulty sleeping. For the diagnosis of AMS, headache is an obligatory criterion with at least one other symptom of the LLS and a total score of ≥3 (Roach et al. 1993). In line with other research a more valid cut-off score in presence of headache is 5 (Kayser et al. 2010; Dellasanta et al. 2007; Wagner et al. 2012).

### Data analyses

Mann–Whitney *U* test was used to calculate group differences. A correlation test by Spearman was applied to calculate correlations between ADACL, VAS and LLS. The statistical program used was SPSS (version 21.0; SPSS Inc., Chicago, USA, 2013). The significance level was set at p < .05.

## Results

All 32 subjects filled in the questionnaires; however, two subjects did not complete the ADACL and were therefore excluded from all analyses concerning the ADACL.

The subjects were divided into an AMS (LLS ≥ 5) and a non AMS group (LLS < 5). Mann–Whitney *U* test calculations showed a significant difference in the VAS items fatigue, drowsiness, alertness and AMS. ADACL, FS and FAS showed no significant differences between the groups (Table 1).

**Table 1:** Mean, standard deviations and group differences between subjects diagnosed with and without AMS by the LSS

Questionnaire	Item	Mean		p
		No AMS	AMS	
		(LLS < 5)	(LLS ≥ 5)	
LLS	Headache	0.19 (±0.40)	2.56 (±0.51)	<.001**
	Gastrointestinal symptoms	0.06 (±0.25)	1.31 (±0.87)	<.001**
	Fatigue and weakness	0.38 (±0.50)	1.19 (±0.91)	.015*
	Dizziness and lightheadedness	0.00 (±0.00)	0.94 (±0.93)	.006**
	Difficulty sleeping	0.63 (±.81)	1.63 (±1.20)	.021*
	Total score	1.25 (±1.24)	7.63 (±1.63)	<.001**
ADACL	Tense arousal	13.75 (±3.04)	16.00 (±6.00)	.448
	Energetic activation	29.63 (±7.81)	25.43 (±8.78)	.093
	Feeling scale	3.31 (±1.78)	2.69 (±1.78)	.305
	Felt arousal scale	2.06 (±1.06)	2.50 (±.97)	.224
VAS	Fatigue	2.50 (±2.13)	4.88 (±2.42)	.004**
	Relaxation	1.69 (±2.44)	2.38 (±2.00)	.184
	Drowsiness	2.31 (±2.09)	5.25 (±2.21)	.001**
	Alertness	2.75 (±2.14)	4.94 (±2.11)	.008**
	Dizziness	.38 (±.72)	1.19 (±1.97)	.445
	Energy	2.69 (±2.73)	4.38 (±2.68)	.061
	AMS	2.19 (±3.49)	4.19 (±2.97)	.039*

The VAS values are given in cm. Data are shown as Mean ± standard deviation for all subjects divided into AMS and non AMS group

*LLS* Lake Louise Self-Report, *ADACL* Activation Deactivation Check List, *VAS* Visual Analogue Scale, *AMS* Acute Mountain Sickness

Significance level: * p < .05; ** p < .01

**Fig. 1:**
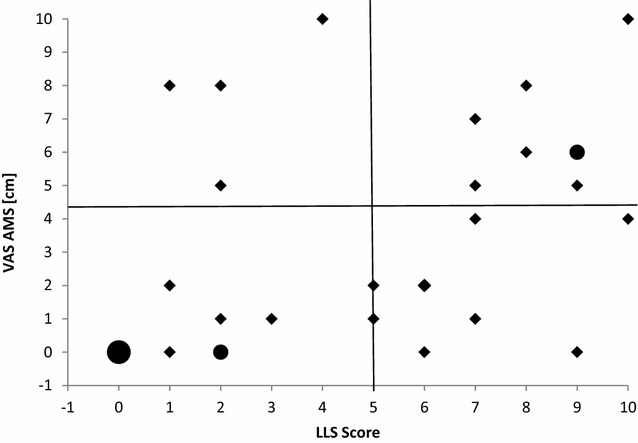
LLS, Lake Louise Self-Report; VAS, Visual Analogue Scale; AMS, acute mountain sickness. Relationship between the LLS score and the single VAS-item AMS. Regression calculations revealed a cut off line for VAS at 4.2 cm for people diagnosed with AMS (LLS ≥ 5). *Black dots* illustrate multiple persons. 6 persons had a LLS and VAS score of 0. *Both other dots* mark two persons

The comparisons between the scores at the different VAS and the LLS score revealed highly significant correlations (p < .001) for six out of seven items (Table 2). The items fatigue, alertness, drowsiness, AMS, energy and the total VAS score showed moderate to strong correlations. Only the item relaxation resulted in a weak correlation with the LLS Score. The item dizziness did not correlate with the LLS score, but instead with the single LLS item dizziness (r = .378, p = .033). Despite the high correlation between the VAS-item acute mountain sickness (AMS) and the LLS score (r = .563, p = .001), no linear correlation was shown (Fig. 1). 50 % of the subjects had a score ≥5 of AMS in LLS. However a significant correlation between the VAS-item AMS and the LLS score was shown for the 50 % of the subjects who scored below 5 (r = .647, p = .007), and for those diagnosed with AMS by the LLS score (≥5) (r = .553, p = .026). The LLS, being predicted from the VAS (LLS ≥ 5; y = 2.789 + .517 *x), revealed a cut-off point of 4.2 cm for the VAS item AMS. Four people diagnosed as not mountain sick by the LLS, listed themselves above the VAS AMS cut-off point. Reversely, eight people who were diagnosed with AMS on behalf of the LLS, stated themselves as not acute mountain sick. 21 people were diagnosed consistently by both scales (8 people with AMS and 13 people without AMS) (Fig. 1).

**Table 2:** Relationship between the LLS score, the single LLS items and the single VAS-items

	VAS fatigue	VAS relaxation	VAS drowsiness	VAS alertness	VAS dizziness	VAS energy	VAS AMS	VAS total
*LLS*								
Total score	.661**	.365*	.700**	.595**	.195	.525**	.563**	.633**
Headache	.454**	.184	.488**	.396*	.087	.275	.424*	.389*
Gastrointestinal symptoms	.590**	.360*	.604**	.598**	.291	.352*	.492**	.563**
Fatigue and weakness	.470**	.362*	.532**	.329	.078	.537**	.414*	.498**
Dizziness and lightheadedness	.457**	.174	.472**	.387*	.365*	.320	.294	.410*
Difficulty sleeping	.347	.193	.409*	.434*	−.026	.376*	.336	.383*

Data are shown as spearman correlation coefficients at one time point at 3650 m (n = 32)

*VAS* Visual Analogue Scale, *VAS AMS* Visual Analogue Score Acute Mountain Sickness, *LLS* Lake Louise Self-Report

Significance level: * p < .05; ** p < .01

**Fig. 2:**
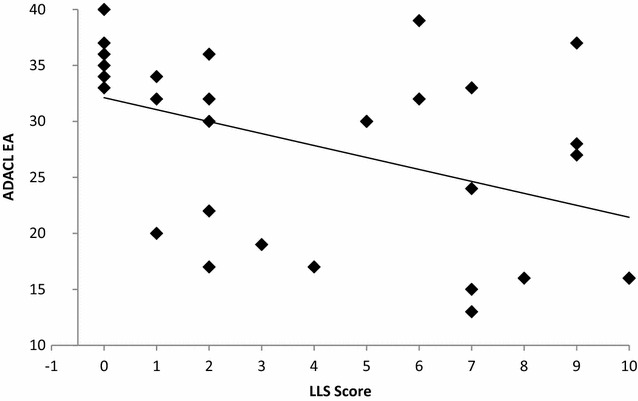
ADACL, Activation Deactivation Check List; LLS, Lake Louise Self-Report. Relationship between the ADACL subscale energetic activation and the LLS score

The ADACL subscale score EA correlated negatively with the LLS score (r = −.536, p < .001) (Table 3; Fig. 2), as well as with the total VAS score (r = −.936, p < .001). On the other hand, no correlation could be shown between the ADACL subscale TA and the LLS as well as the FS (r = −.328, p = .068). A negative correlation was found for the FS and the VAS-item AMS (r = −.535, p = .002), the total VAS score (r = −.610, p < .001) and the ADACL subscale EA (r = .683, p < .001). In people diagnosed with AMS (LLS ≥ 5), FS showed only a significant negative correlation with the VAS item energy (r = −.645, p = .007). The FAS showed no relationship to any scores despite the ADACL subscale tension state (r = .461, p = .010).

**Table 3:** Relationship between the LLS score and the ADACL subscales, FS and FAS

	ADACL [EA]	ADACL [TA]	FS	FAS
LLS score	−.536**	.190	−.347	.202

Data are shown as spearman correlation coefficients at one time point at 3650 m. N = 32 for FS and FAS, n = 30 for ADACL EA and TA

*LLS* Lake Louise Self-Report, *ADACL [EA]* Activation Deactivation Check List Energetic Activation, *ADACL [TA]* Activation Deactivation Check List Tense Arousal; FS, Feelings Scale, *FAS* Felt Arousal Scale

Significance level: * p < .05; ** p < .01

## Discussion

In line with other research (Hext et al. 2011; Kayser et al. 2010; Dellasanta et al. 2007; Wagner et al. 2007, 2012), the present study showed correlations between the items of the VAS and the LLS score. In previous research no comparisons were made between single VAS-items asking about AMS symptoms and the total LLS score (Hext et al. 2011; Wagner et al. 2007, 2012). Only Kayser et al. (2010) plotted the VAS-item headache versus the LLS-item headache, and the data points did not conform to the line of identity. The VAS item headache was not included in the present study, nevertheless moderate to strong correlations between the single VAS-items and the total LLS score were found for 6 out of 7 items. Only the item dizziness was not related to the LLS score, but to the LLS item dizziness. Out of sixteen people who had a LLS Score of 5 or above, only six reported moderate dizziness. Further, the VAS Score dizziness showed no significant difference between those diagnosed with AMS (LLS ≥ 5) and those who had a LLS Score below 5. This raises the question if dizziness is a necessary item in assessing AMS. All subjects reporting dizziness and lightheadedness had an LLS Score above 7. Thus the item did not contribute to the cut-off of a LLS-score of 5. Out of 7 items of the VAS-score, 4 showed a significant difference between the AMS groups. Those were namely fatigue, alertness, drowsiness and AMS. People who were assessed as acute mountain sick by the LLS defined themselves significantly different with AMS on the VAS scale (Table 1).

The linear scaling characteristics of the VAS item AMS and the LLS score (Fig. 1) do not plot close to a theoretical identity line. Kayser et al. (2010) revealed in their study a threshold effect for the LLS scores below 5–6 leading to low VAS scores. The present study does not reflect this result. Nevertheless only 64 % of the subjects were consistently diagnosed with AMS by both scales. Due to the VAS-AMS-scale, four subjects who would not have been diagnosed with AMS by the LLS (score < 5) described themselves as moderately to almost severely mountain sick. Eight people who would have been diagnosed as sick by the LLS (score ≥ 5) were below the cut off line of the VAS. In regard to previous literature (Hext et al. 2011; Wagner et al. 2007) and the results of the actual study, we think that a single VAS-item to assess overall AMS intensity can help to immediately assess the changes in the sensation of AMS but might only be effective for experienced mountaineers. However, moderate to strong correlations between the VAS and the LLS have been shown. Therefore, to answer our first research question, we found a strong relationship between the VAS measurement and the LLS. Despite the huge reputation of the LLS, in our opinion a VAS system might give further possibilities to work out a more valid cut off-score in comparison to an instrument with a 4-point Likert approach as the LLS, in the future.

The second research question involves a possible relationship between psychological well-being and AMS. Due to the strong relation between the VAS items relaxation, drowsiness, alertness, energy and the ADACL subscale EA with the LLS (Fig. 2), AMS might be related to a greater complex of physiological and psychological well-being. Moreover, a significant difference in the ADACL subscale EA was calculated for people diagnosed with AMS (LLS ≥ 3). People suffering from AMS felt less energetic than healthy subjects. The subscale EA covers items including fatigue and alertness. These items were also part of the VAS and thus showed a strong relationship in direct comparison. Despite this relationship, ADACL might not be a good tool for measuring AMS because it takes a long time to fill in the 20 items of this instrument and furthermore, subscales have to be calculated afterwards. A shorter way of assessing subjective mood is the FS. It showed a moderate negative effect (r = −.536) for the VAS-item AMS and every other item of the VAS, but no correlation to the LLS score, or any items of the LLS. One might assume that subjective well-being diminishes when people suffer from AMS. Nevertheless in the study from Shukitt-Hale et al. (1991) happiness improved at a height of 4300 m even though the people became less friendly, less clear thinking, sleepier and dizzier. If the LLS remains the most used questionnaire for assessing AMS, it could be concluded that the subjective well-being might not be a factor for measuring AMS, but nevertheless could influence it. In people diagnosed with AMS (LLS ≥ 5) the subjective well-being is negatively correlated with energy but not with AMS. In climbers who started to experience either fatigue or AMS at a height of 5640, subjective vitality and intrinsic motivation scores dropped (Norling et al. 2014). Therefore, research in this field might contribute to more knowledge of potential interactions between psychological states and AMS. Furthermore research has to examine how a reduced psychological well-being might be a predictor or an early warning for AMS.

Limitations include slightly different weather conditions between the measurements in October and May. This could be a factor which may have affected psychological well-being of participants. Due to the non-existence in the LARS (Gudgeon and Hindmarch 1983), the present study did not contain the VAS-item headache, which might be discussed as a limitation of this approach. Furthermore, some of the subjects were already accustomed to the questionnaires because of former high altitude study participation in a laboratory situation. As a methodical limitation of this study it should be discussed that AMS was defined with an LLS ≥ 5. The discussed results of the VAS are therefore linked to the LLS. Furthermore, no tests for sensitivity and specificity as well as no decision plots for the determination of optimum values of VAS for the diagnosis of AMS as it was done by Slingo et al. (2012), have been performed in this study due to a low number of repeated measurements.

In conclusion, this study showed moderate to strong correlations between the VAS-items and the LLS. Using the single VAS-item AMS can be a helpful alternative to questionnaires like the LLS. Changes in AMS might be detected more specifically and thus the decision on the descent of a person with AMS could be taken more accurately. A reduced subjective well-being resulted in reduced energy and vice versa in people diagnosed with AMS. Correlations between AMS and subjective well-being could not been shown in this study. Future studies should try to assess physiological as well as psychological predictors of AMS and compare it to the VAS item AMS and the LLS.
